# Cross-cultural translation, adaptation, and validation of the Amharic version pain self-efficacy questionnaire in people with low back pain in Ethiopia

**DOI:** 10.1186/s12891-021-03985-4

**Published:** 2021-01-25

**Authors:** Mulugeta Bayisa Chala, Catherine Donnelly, Yemataw Wondie, Setareh Ghahari, Jordan Miller

**Affiliations:** 1 Queen’s University, School of Rehabilitation Therapy, Kingston, ON Canada; 2 Department of Physiotherapy, University of Gondar, College of Medicine and Health Sciences, Gondar, Ethiopia; 3 Department of Psychology, University of Gondar, College of social Sciences and Humanities, Gondar, Ethiopia

**Keywords:** Low back pain, Self-efficacy, Ethiopia, Reliability, Validity, Cross-cultural translation, Cross-cultural adaptation

## Abstract

**Background:**

The Pain Self-Efficacy Questionnaire (PSEQ) is a valid and reliable instrument that evaluates pain self-efficacy beliefs in people with pain conditions. However, it has not been validated and used in Ethiopia. We conducted this study to translate, adapt, and test the psychometric properties of the PSEQ in the Amharic language and Ethiopian context for its use with people experiencing low back pain (LBP).

**Methods:**

The PSEQ was translated into Amharic and then back-translated into English. An expert review committee created a final Amharic version of the tool (PSEQ-Am), followed by pilot testing and cognitive debriefing with a sample of 20 people with LBP. The psychometric properties of the final version of PSEQ-Am were assessed in a sample of 240 people with LBP recruited from three rehabilitation centers in Ethiopia. Cronbach’s alpha and Intra-class correlation coefficient were calculated to describe the reliability and internal consistency of the tool. The SF-36-Am bodily pain subscale was used to assess convergent validity. Confirmatory Factor Analysis (CFA) and Exploratory Factor Analysis (EFA) were performed to determine the dimensionality of the instrument.

**Results:**

PSEQ-Am demonstrated excellent test-retest reliability (ICC = 0.93) and internal consistency (Cronbach’s alpha = 0.91). As hypothesized, the tool demonstrated a significant moderate correlation with the Bodily Pain subscale of the SF-36-Am (Rho = 0.51, *p* < 0.01). EFA analysis shows that the Amharic version of PSEQ is a dominant one factor and secondary two factor structure.

**Conclusion:**

This study shows that PSEQ-Am is a reliable and valid tool that can be used in both clinical practice and research in the Ethiopian low back pain population.

**Supplementary Information:**

The online version contains supplementary material available at 10.1186/s12891-021-03985-4.

## Background

Low back pain (LBP) and its associated disability is an important global public health problem [1, 2]. While the majority of LBP episodes resolve in a few weeks, a minority of people with LBP suffer from recurrent and chronic pain [3].

LBP is a complex phenomenon that occurs as a result of the interaction of biological, psychological, and social factors [4]. Pain is also an experience that may challenge one’s beliefs about their ability to perform certain activities of daily living [5]. Certain psychological factors are significant predictors of disability and recovery among people with LBP [6–8]. For instance, previous research highlights that poor perception of personal control and low confidence in the ability to perform certain activities in spite of pain were predictors of poor treatment outcomes among people with chronic LBP [9–11]. Similarly, an increase in self-efficacy beliefs during treatment of LBP is associated with better long-term outcomes, such as improved physical functioning and self-reported pain at 6 months [12].

Albert Bandura stated that perceived self-efficacy is “the conviction that one can successfully execute the behavior required to produce the outcomes” (Bandura 1977, P.193) [13]. According to Bandura, higher self-efficacy enhances human accomplishment by making them approach difficult tasks to be mastered rather than threats to be avoided [14]. In addition, self-efficacy determines whether or not people will persist in the face of obstacles and their motivation to change their circumstances [14, 15]. In contrast, people with low self-efficacy shy away from approaching difficult tasks and show weak commitment to the goal they want to pursue [12].

Higher self-efficacy is hypothesized as a protective factor which helps in the adjustment to living with chronic pain [16]. Evidence also suggests that self-efficacy mediates the impact of pain on disability [17] and influences one’s readiness to engage in pain coping strategies [18]. For instance, patients with low self-efficacy are more likely to have catastrophic thoughts related to their pain and often use more passive coping mechanisms instead of engaging in active coping mechanisms [19]. LBP management approaches have increasingly incorporated cognitive-behavioral strategies to change the way patients think about their pain-related disability and challenge their beliefs about personal control related to activities and functioning [9, 12]. Given the mediator role of self-efficacy between pain and disability, it suggested that health professionals working with people experiencing LBP target enhancing patient’s self-efficacy as one of the rehabilitation goals [12, 19, 20].

The Pain Self-Efficacy Questionnaire (PSEQ) is a brief, yet comprehensive tool which assesses different dimensions of patient’s self-efficacy related to physical functioning, social interaction, and participation in valued activities of daily living in the presence of pain [6, 15]. This tool has been validated and used among people with different pain conditions including LBP in a number of settings and different cultures [6, 8, 21–26]. Assessing self-efficacy is important in pain research and rehabilitation [6, 12, 20, 23, 27]. This construct is well investigated among people with LBP in economically advanced countries [25], but self-efficacy has not been extensively explored in low-income countries like Ethiopia. Lack of validated measures of self-efficacy may be among the reasons why the self-efficacy construct has not been well addressed in research and clinical practice in these countries.

Therefore, this research aims to translate and adapt PSEQ into Amharic and to evaluate its psychometric properties, including internal consistency, test-retest reliability, convergent construct validity, and factor structure among Ethiopian people with LBP.

## Methods

To meet the study objectives, this study was completed in two phases. Phase 1 was to translate and culturally adapt the PSEQ to the Amharic language. Phase 2 involved testing psychometric properties of the tool. Details of each phase are included below. Permission to cross-culturally translate, adapt, and validate PSEQ into the Ethiopian context was obtained from the original tool developer (Michael Nicholas) [15].

### Phase 1 - translation and cultural adaptation into the Amharic language

The translation and adaption of PSEQ to Amharic language was conducted according to a five-step process suggested in a guideline by Beaton et al. (2000) for cross-cultural adaptation of self-report measures [28].

In **Step 1**, two translators (both physiotherapists) who are fluent in both Amharic and English languages independently translated the original PSEQ into Amharic language (PSEQ-Am). In **Step 2**, the independently translated documents (T1) were shared among the two translators to synthesize each other’s translation to produce Amharic version PSEQ1. Any differences in the meaning and concepts of the translation were discussed and resolved via consensus. **Step 3** involved the back translation of the first draft (PSEQ1) to English. This was performed by two independent translators [one who is a clinical Psychologist and another who is a professor of Teaching English as a Foreign Language at the University of Gondar]. One of the translators (professor of Teaching English as a Foreign Language) was unfamiliar with the construct being assessed and PSEQ measure. The other translator was familiar with the construct being assessed. A third back translation was created through a discussion between the two back translators, facilitated by the lead author, who reached agreement on an English back translation. All three documents were provided to the panel for step 4.

In **Step 4**, a panel of experts (all four translators, a research methodologist from University of Gondar Institute of Public Health, a neurosurgeon at the University of Gondar Comprehensive Specialized Hospital, and a native English speaker who is also fluent in the Amharic language) was assembled to review and synthesize every step of the translation and adaptation process and verify the tool for pre-field testing. Next, the questionnaire was piloted with 20 people with LBP who were receiving physiotherapy services at the University of Gondar comprehensive specialized hospital. A qualitative cognitive debriefing [29] was conducted with the 20 participants to assess the clarity of the instruction, understandability of the items and language, and cultural appropriateness and acceptability of the PSEQ-Am (**Step 5)**. After the five steps, the translated and adapted PSEQ-Am was ready for psychometric testing.

### Phase 2 - psychometric testing of the PSEQ-am

#### Participants

To assess the psychometric properties of PSEQ-Am, a convenience sample of 240 people with LBP were recruited from three referral hospitals in Ethiopia: Physiotherapy Departments of the University of Gondar Comprehensive Specialized Hospital, Bahirdar Felege Hiwot Comprehensive Specialized hospital, and Black Lion hospital in Ethiopia. The present sample size fulfills the requirement for survey tool validation (e.g., 5–10 participants per item in the tool) [26, 30]. Individuals who: were ≥ 18 years of age, had non-specific LBP of any duration, were willing to consent and participate in the study, and self-reported the ability to understand and speak Amharic language were included in this study. Exclusion criteria were pregnancy, people with cancer-related pain, and people with LBP visiting the hospitals primarily for other health problems.

### Patient-reported measures

*Pain Self-Efficacy Questionnaire (PSEQ)* - The PSEQ is a 10-item self-reported tool intended to measure patients’ belief in their abilities to perform a number of activities despite pain [15]. Each item is rated on a 7-point Likert Scale (0- not confident at all and 6-completely confident) to yield a total score of 0 to 60 after the scores of each item are summed [15]. A higher score indicates greater self-efficacy in performing certain activities despite pain [15]. PSEQ has demonstrated strong psychometric properties such as high internal consistency, a high degree of stability, and construct validity in both the original version among people with heterogeneous chronic pain problems [15] and among people with LBP [21, 27, 31].

*Short Form Health Survey (SF-36)* – The SF-36 is a health status questionnaire that has frequently been used to assess the convergent validity of patient-reported outcome measures, including self-efficacy tools [22, 32–37]. The SF-36 has 36 items intended to measure general health status and quality of life of people across a range of medical conditions [38, 39]. The tool assesses 8 major domains across physical and mental health [39, 40]. For each of the 8 domains, the items were coded, summed, and transformed into a 0–100 scale, where 0 indicates the worst health-related quality of life and a score of 100 indicates the best health-related quality of life [33, 36]. The bodily pain subscale was reverse coded before transforming into a 0–100 scale to ensure that the higher item value indicates better health on SF-36-Am [41]. The SF-36 has been translated into Amharic (SF-36-Am) and the SF-36-Am has been psychometrically tested and shown to have acceptable internal consistency and construct validity among the general population in Ethiopia [42].

### Reliability

The reliability of the PSEQ-Am was established in two ways. First, Cronbach’s alpha was calculated to assess the internal consistency of the tool. An alpha value of > 0.7 was considered acceptable, > 0.8 was considered good, and > 0.9 excellent [43]. We also conducted a sub-group analysis of the tool’s internal consistency by sex of the participants and the duration of pain: acute vs. chronic pain (pain lasting > 3 months).

Second, the test-retest reliability of the Amharic PSEQ was examined. A third of the participants (80 individuals with LBP) were asked to complete the PSEQ-Am a second time between 3 and 5 days after they completed it the first time [21]. This period was determined to accommodate people with LBP who come from rural areas as they may not be able to stay in the cities for an extended period to receive rehabilitation services. It is also expected that the clinical condition of people with LBP would likely remain stable during this period [44].

### Convergent construct validity

To test the convergent construct validity of PSEQ-Am, we performed correlation tests between the tool’s total score and SF-36-Am subscales. Previous studies indicated the presence of a moderate positive correlation between the PSEQ total score and the Bodily Pain subscale of the SF-36 [8, 22]. Since PSEQ is a measure of patients’ self-efficacy beliefs in executing various daily living activities by taking pain into consideration [15], we also expected to see a moderate positive correlation with the Bodily Pain subscale of the SF-36-Am. Furthermore, we expected positive but weak to moderate correlations between PSEQ-Am and the rest of SF-36-Am subscales, similar to the findings reported in previous studies [8, 22, 25]. Construct validity for PSEQ-Am was considered adequate when > 75% of a priori hypothesis was met [45, 46]. Furthermore, we hypothesized that PSEQ-Am would demonstrate a unidimensional construct in a factor analysis similar to what was reported in previous studies [15, 22].

#### Data collection process

Data were collected by 3 trained physiotherapists who were working at the study sites. Written informed consent was obtained from each participant who was willing to partake in the study. Socio-demographic information, PSEQ-Am, and SF-36-Am questionnaires were provided and the questions were read aloud to participants. Clinical information including age, diagnosis, and duration of pain were extracted from the participant’s medical chart using a data abstraction tool. Data were collected from April 7 to August 5, 2019.

### Analysis

Statistical analyses were conducted using Statistical Package for Social Sciences version (IBM SPSS statistics 25. Inc). Demographic information was described using means and standard deviations or medians and interquartile range for continuous data; and counts and percentages for categorical data. Intraclass-correlation coefficient (ICC_agreement_ 2,1) and Cronbach’s alpha were calculated to assess the test-retest reliability and internal consistency, respectively. The ICC_agreement,_ 2,1 was calculated based on a two-way random effects model [47, 48]. A Bland-Altman plot of differences was also conducted to evaluate test-retest reliability [45, 46]. The Bland-Altman 95% Limit of Agreement (LOA) indicates the magnitude of random changes caused by systematic variation or random measurement error [49].

The plan to calculate the correlation between Amharic versions PSEQ and SF-36 items was to use Pearson Correlation coefficients if data met all assumptions, including normality and to use Spearman’s Correlation coefficients (Rho) if the assumption of normality was not met [50]. The normality of data distribution was checked using the Shapiro-Wilk test of normality, and by assessing Skewness and Kurtosis coefficients [51]. PSEQ-Am did not demonstrate normality of distribution with the Shapiro-Wilk test of 0.94 (*p* < 0.05), and Skewness (− 0.76) and Kurtosis (0.35) coefficients. Hence, Spearman’s correlation (Rho) was performed to explore the convergent construct validity of the PSEQ-Am. Rho values < 0.4, 0.4–0.69, and ≥ 0.7 were considered weak, moderate, and strong correlation, respectively [52, 53]. We conducted a disaggregated analysis of the correlation to investigate the differences between Male and Female [54] and between the acute and chronic (pain lasting more than 3 months) samples.

The floor and ceiling effect of PSEQ-Am was established by calculating the percentage of respondents who scored the lowest or highest possible scores on the tool [45].

### Factorial validity

Confirmatory Factor Analysis (CFA) was performed to assess a previously hypothesized one-factor solution [15] for the PSEQ-Am using analysis of Moment Structures (IBM AMOS version 25) software. The quality of model fitness was determined using several fit indices and their criteria such as Chi-square (X^2^/df), Comparative Fit Index (CFI), Goodness of Fit Index (GFI), Parsimony Goodness of Fit Index (PGFI), and Root Mean Square Error of Approximation (RMSEA) [55]. A model for one-factor solution was considered acceptable if X^2^/df is 5 [56], CFI and GFI > 0.8 [55], PCFI and PGFI > 0.6 [57], and RMSEA is < 0.08 [58]. A model was considered a good fit if X^2^/df is 2 [56], CFI and GFI > 0.9 [55], PCFI and PGFI > 0.8 [57], and RMSEA is < 0.05 [19, 58].

## Results

### Phase 1 - cross-cultural translation and adaption

The translators and the panel of experts reached an agreement during the translation and adaption of the PSEQ into the Ethiopian context. None of the items in the original tool were deleted in either forward or backward translations. However, item #2 [I can do most of the household chores (e.g., tidying -up, washing dishes, etc.), despite the pain] and item #10 [I can gradually become more active, despite pain] were adapted to the local culture and context to maintain conceptual equivalence, and increase participants comprehension of the items.

Changes to item #10 were made by the translators (T1 and T2). The literal translation of item #10 “I can gradually become more active, despite pain” to Amharic would indicate someone’s level of alertness or consciousness, which can deviate from the conceptual meaning of an original tool. Since the direct translation of “active” into Amharic language is broad and can result in a different interpretation of the item, a modification was made to increase the conceptual equivalence of the tool by the participants. Hence, the translators (T1 and T2) suggested the item should be modified accordingly, “I can gradually become more *physically* active, despite the pain.”

The socio-demographic and clinical characteristics of the sample (*n* = 20) for the pilot testing and cognitive debriefing of the pre-final Amharic PSEQ are described in Table 1. None of the participants reported difficulty in understanding and completing the tool during the cognitive debriefing**.** They also re-iterated that the items in the tool are well organized, easy to understand, direct, concise and unambiguous, and culturally acceptable. A modification was made to item #2 in response to the cognitive debriefing. Item #2 states: “I can do most of the household chores (e.g., tidying -up, washing dishes, etc.), despite the pain”. Some of the men participating in the cognitive interview suggested the examples provided tended to be performed by women in Ethiopia and thought examples that are traditionally done by men would help men to answer this question. The participants suggested activities such as making a bed, cleaning the house, picking up things, splitting firewood, and cooking a meal to provide a broader list of items including those performed by men and women. Based on the participants’ feedback, the committee modified item #2 as “I can do most of the household chores (e.g., cleaning the house, washing dishes, making a bed, picking things up, cooking a meal, splitting firewood, etc.) despite the pain.”

**Table 1:** socio-demographic and clinical characteristics of participants who participated in the pilot and cognitive debriefing study

Characteristics		N (%)	Mean (SD)
Age			36.3 (15.5)
Sex	Male	8 (40%)	
	Female	12 (60%)	
Educational status	Uneducated	2 (10%)	
	Primary School	6 (30%)	
	Secondary School	4 (20%)	
	Tertiary School (College or University)	8 (40%)	
Residence	Urban	12 (60%)	
	Rural	8 (40%)	
Duration of pain (in months)			14.5(16.1)

Finally, four participants recommended that every number on the scale should have descriptors. The original PSEQ has anchors at 0 (“Not confident at all,”) and 6 (“Completely confident”). The respondents advised that every number in the scale (1–5) should have worded anchors to make it easy for the respondents to choose their responses accurately. Although we acknowledge the respondent’s feedback valuable, the panel of experts agreed that making such modification on the scale may impact the performance of the scale, and therefore, no descriptors were added.

The final version of the PSEQ-Am is available in appendix 1.

### Phase 2 - psychometric testing

#### Demographics and clinical characteristics of the study participants

Two hundred and forty people (52 acute and 188 chronic) with LBP participated in this validation study. The participants’ mean age was 40.93 (SD = 13.5) years old. Most of the participants were women (59.2%), married (59.2%), and had attended a tertiary level (college or university) education (39.2%) (see Table 2 for demographic information). The mean duration of pain was 41.65 (SD = 51.87) months. Most of the participants (*n* = 188,78.3%) had chronic LBP. The median and Interquartile range of PSEQ-Am scores were 46 and 18, respectively. A total of 186 (77.6%) of study participants received some forms of pain medication, physiotherapy, or a combination of both for their pain prior to their participation in this study (see Table 2 for clinical characteristics of the study participants).

**Table 2:** The socio-demographic and clinical characteristics of people with LBP at University of Gondar Comprehensive Specialized Hospital, Bahirdar Felege Hiwot Comprehensive Specialized Hospital, and Black Lion Hospital, Ethiopia

Characteristics ^b^		Acute (***n*** = 52)	Chronic (***n*** = 188)	Total (***n*** = 240)
		n (%)	n (%)	n (%)
Mean (SD) age in years		33.4 (9.92)	43 (13.69)	40.93 (13.5)
Sex	Male	25 (48.1%)	73 (38.8%)	98 (40.8%)
	Female	27 (51.9%)	115 (61.2%)	142 (59.2%)
Marital status	Single	19 (36.5%)	46 (24.5%)	65 (27.1%)
	Married	29 (55.8%)	113 (60.1%)	142 (59.2%)
	Cohabited	0 (0%)	10 (5.3%)	10 (4.2%)
	Divorced	2 (3.8%)	14 (7.4%)	16 (6.7%)
	Widowed	2 (3.8%)	5 (2.7%)	7 (2.9%)
Educational status/level	Uneducated	1 (1.9%)	17 (9.0%)	18 (7.5%)
	Primary School	10 (19.2%)	35 (18.6%)	45 (18.8%)
	Secondary School	18 (34.6%)	65 (34.6%)	83 (34.6%)
	Tertiary School (College or University)	23 (44.2%)	71 (37.8%)	94 (39.2%)
Residence	Rural	7 (13.5%)	19 (10.1%)	26 (10.8%)
	Urban	45 (86.5%)	169 (89.9%)	214 (89.2%)
Religion	Orthodox Christian	36 (69.2%)	158 (84.0%	194 (80.8%)
	Muslim	9 (17.3%)	18 (9.6%)	27 (11.3%)
	Protestant	6 (11.5%)	10 (5.3%)	16 (6.7%)
	Atheist	0 (%)	1 (0.5%)	1 (0.40%)
	Others	1 (1.9%)	1 (0.5)	2 (0.80%)
Employment / work status	Employed (government or private)	29 (55.8%)	102 (54.3%)	131 (54.6%)
	Farmer	3 (5.8%)	9 (4.8%)	12 (5%)
	Housewife	8 (15.4%)	36 (19.1%)	44 (18.3%)
	Student	7 (13.5%)	8 (4.3%)	15 (6.3%)
	Retired	5 (9.6%)	13 (6.9%)	18 (7.5%)
	Others	0 (0%)	20 (10.6%)	20 (8.3%)
Diagnosis provided on patient’s medical chart	No clear diagnosis provided	28 (53.8%)	91 (48.4%)	119 (49.6%)
	LBP secondary to disc prolapse	15 (28.8%)	54 (28.7%)	69 (28.8%)
	LBP secondary to degenerative changes	6 (11.5%)	13 (6.9)	33 (13.8%)
	Other causes	3 (5.8%)	30 (16%)	19 (7.9%)
Pain management documented on medical charts	A combination of physiotherapy and pain killers	15 (28.8%)	60 (31.9%)	75 (31.3%)
	Pain killers only	13 (25%)	39 (20.7%)	52 (21.7%)
	Physiotherapy only	9 (17.3%)	50 (26.6)	59 (24.6%)
	None	15 (28.8%)	39 (20.7%)	54 (22.5%)
Mean (SD) duration of pain in months		1.63 (0.67)	52.7 (53.6)	41.7 (51.8)
Mean Pain Self-Efficacy Questionnaire (0–60) (SD)		40.5 (13.5)	44.8 (11.5)	43.8 (12)
SF-36 (0–100) ^a^				
Physical functioning (PF)^c^		57.5 (35)	55 (35)	55 (35)
Role Functioning Physical (RP)^c^		25 (25)	25 (50)	25 (50)
Role Functioning Emotional (RE)^c^		0 (33)	0 (33)	0 (100)
Energy (VT) ^c^		65 (23.8)	60 (20)	60 (20)
Emotional Well-Being (EW) ^c^		68 (23)	64 (24)	68 (84)
Social Functioning (SF) ^c^		75 (34.4)	75 (25)	75 (25)
Bodily Pain (BP) ^c^		32.5 (30.4)	45 (35)	37.5 (35)
General health perception (GH) ^c^		60 (23.8)	50 (25)	55(25)

^a^one participant (out of the total 240) did not complete the SF-36 questionnaire

^b^ each column presents frequencies and percentages except where indicated

^c^Median and Interquartile range

#### Reliability of the PSEQ-am

The Amharic version of PSEQ demonstrated excellent internal consistency with a Cronbach’s alpha value of 0.90. In addition, the internal consistency of the tool if a single item is deleted range from 0.88–0.90, which is comparable to the overall alpha value (see Table 3). PSEQ-Am also demonstrated an excellent level of reliability in the subgroup analysis by sex (Male: α = 0.90 vs. Female: α =0.90) and by the duration of pain (Acute LBP: α = 0.89 vs. Chronic LBP: α =0.90).

**Table 3:** The internal consistency of PSEQ-Am if item deleted (*N* = 239)

Item-Total Statistics					
	Scale Mean if Item Deleted	Scale Variance if Item Deleted	Corrected Item-Total Correlation	Squared Multiple Correlation	Cronbach’s Alpha if Item Deleted
PSEQ1	39.44	125.059	.492	.317	.904
PSEQ2	40.01	116.653	.638	.601	.895
PSEQ3	39.10	121.736	.627	.509	.896
PSEQ4	39.47	119.162	.687	.502	.892
PSEQ5	39.64	114.356	.769	.715	.886
PSEQ6	39.51	113.573	.736	.601	.889
PSEQ7	40.18	114.229	.602	.466	.900
PSEQ8	39.24	117.598	.740	.669	.889
PSEQ9	39.09	118.825	.715	.703	.891
PSEQ10	39.06	122.499	.624	.560	.896

**Fig. 1:**
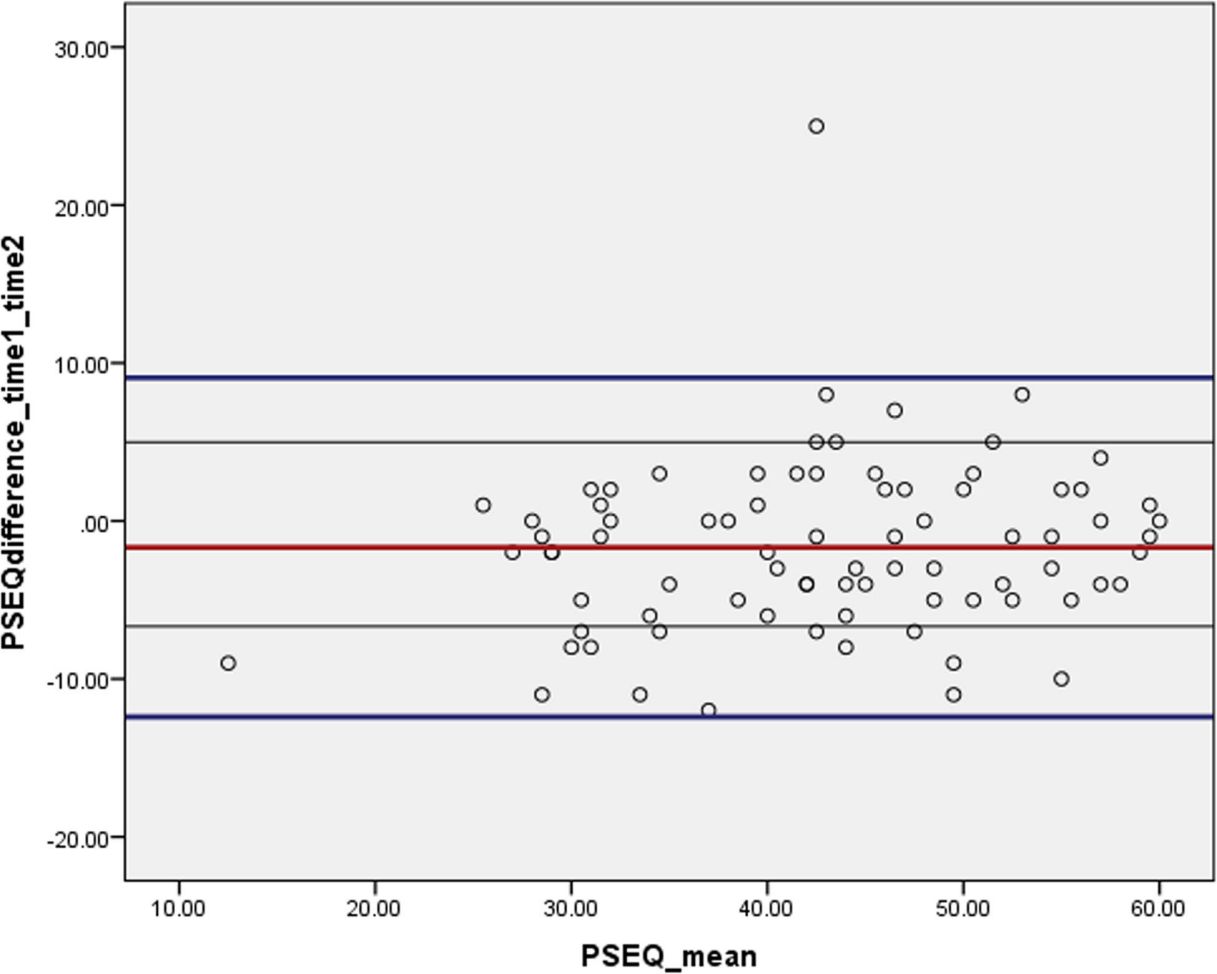
Bland-Altman plot of agreement between test and re-test scores of the PSEQ-Am with a red line representing the mean of the difference, bold blue lines representing the 95% limits of agreement (LOA) and normal black lines representing the 95% confidence interval (CI) of the mean of the difference

All 80 participants who were invited to fill out the questionnaire at a second time-point (3–5 days later) completed the questionnaire. The test-retest intra-correlation coefficient (ICC) value was 0.93 (95% CI: 0.88 to 0.95), indicating excellent test-retest reliability. In addition, the Bland-Altman plot indicates that all observation except one, gathered near zero line within the limit of agreement (LOA) ±1.96 SD = ±5.47, further confirming the tools test-retest reliability. The result of the Bland-Altman analysis shows no evidence of systematic change between the two scores, which suggests the stability of the tool over time. The 95% LOA between the two test duration ranged between − 12.41 and 9.06 (Fig. 1).

#### Floor and ceiling effect

One (0.4%) and eleven (4.6%) of the participants scored a total score of 1 and 60 on PSEQ-Am, respectively.

#### Convergent construct validity

Out of the total sample, 239 participants completed both PSEQ-Am and SF-36-Am measures, with all items completed in the questionnaires. One participant who was in severe pain during the time of the interview did not complete the SF-36 questionnaire. As hypothesized, the tool demonstrated a moderate positive correlation with the Bodily Pain (Rho = 0.51, *p* < 0.01) and the General Health Perception (Rho = 0.40, *p* < 0.01) subscales of the SF-36-Am among the whole sample (*n* = 239). PSEQ-Am also showed positive but weak correlations with the remaining SF-36-Am subscales (see Table 4). Considering > 75% of a priori hypotheses were met, PSEQ-Am is considered to have high convergent construct validity (Table 5).

**Table 4:** Spearman’s rho correlation coefficient of the sum score of PSEQ-Am with the sub-scale of the SF-36 (*N* = 239)

SF-36	Correlation with total score of PSEQ-Am (95% CI) (***n*** = 239)	Correlation by duration of pain (95% CI)		Correlation by sex of the participants (95% CI)	
		Acute LBP (***n*** = 52)	Chronic LBP (***n*** = 187)	Male (***n*** = 97)	Female (***n*** = 142)
Physical functioning (PF)	0.38 (0.26–0.48) **	0.22 (−0.10–0.52)	0.44 (0.30–0.56) **	0.48 (0.30–0.63) **	0.32 (0.14–0.48) **
Role Functioning Physical (RP)	0.32 (0.19–0.43) **	0.52 (0.28–0.70) **	0.24 (0.13–0.40) **	0.37 (0.18–0.54) **	0.27 (0.09–0.42) **
Social Functioning (SF)	0.36 (0.25–0.47) **	0.45 (0.21–0.65**)	0.35 (0.21–0.50) **	0.46 (0.23–0.62) **	0.28 (0.13–0.44) **
Bodily pain (BP)	0.51 (0.40–0.61) **	0.67 (0.47–0.80) **	0.46 (0.33–0.56) **	0.55 (0.37–0.68) **	0.47 (0.32–0.60) **
Role Functioning Emotional (RE)	0.28 (0.17–0.4) **	0.22 (−0.02–0.43)	0.29 (0.15–0.42) **	0.36 (0.16–0.53) **	0.21 (0.05–0.36) **
Emotional Well-Being (EW)	0.36** (0.23–0.47)	0.23 (0.16–0.67)	0.42**(0.23–0.53)	0.55 (0.40–0.68) **	0.24 (0.08–0.40) **
Energy /Vitality (VT)	0.31** (0.20–0.42)	0.17 (−0.07–0.44)	0.36 (0.22–0.49) **	0.39 (0.18–0.57) **	0.24 (0.06–0.40) **
General health perception (GH)	0.40** (0.28–0.52)	0.36 (0.10–0.58) **	0.45 (0.32–0.56) **	0.44 (0.23–0.60) **	0.37 (0.20–0.51) **

*PSEQ* Pain Self-Efficacy Questionnaire, *SF-36* short form Medical outcome survey, *LBP* Low back pain

**. correlation is significant at 0.01 significant level (2-tailed), CI: Confidence Interval

**Table 5:** A priori hypothesis to assess convergent construct validity between PSEQ-Am and SF-36-Am subscales in Ethiopian patients with LBP based on COSMIN guideline (*n* = 239)

Outcomes	Hypothesized correlations^a^	Estimated correlations^a^	Hypothesis acceptance (Yes or No)
**SF-36-Am subscales**			
Physical functioning (PF)	0.00–0.69	0.38	Yes
Role Functioning Physical (RP)	0.00–0.69	0.32	Yes
Social Functioning (SF)	0.00–0.69	0.36	Yes
Bodily pain (BP)	0.40–0.69	0.51	Yes
Role Functioning Emotional (RE)	0.00–0.69	0.28	Yes
Emotional Well-Being (EW)	0.00–0.69	0.36	Yes
Energy /Vitality (VT)	0.00–0.69	0.31	Yes
General health perception (GH)	0.00–0.69	0.40	Yes
**Number of met hypotheses (%)**	**8/8 (100)**		

^a^ positive correlation, *SF-36-Am* Amharic version of the short form health survey, *PSEQ-Am* Amharic version of the Pain Self-efficacy Questionnaire

The subgroup analysis by the duration of pain indicated that the PSEQ-Am demonstrated a positive, moderate correlation with four subscales of the SF-36-Am among people with chronic LBP (*n* = 187): Bodily Pain (Rho = 0.46, *p* < 0.01), Physical Functioning (Rho = 0.44, *p* < 0.01), Emotional Well-being (Rho = 0.42, *p* < 0.01), and General Health Perception (Rho = 0.45, *p* < 0.01). For people with acute low back pain (*n* = 52), there was a moderate correlation between the PSEQ-Am and three of the SF-36 subscales: Bodily Pain (Rho = 0.67, *p* < 0.01), Role Functioning Physical (Rho = 0.52, *p* < 0.01), and Social Functioning (Rho = 0.45, *p* < 0.01). Table 4 presents the convergent validity of the Amharic version PSEQ as compared to the summary scores of SF-36 by the duration of pain.

A further disaggregate analysis by sex shows that PSEQ-Am demonstrated a moderate positive correlation with the Bodily Pain sub-scale of SF-36-Am in both Male (Rho = 0.55, *p* < 0.01) and Female (Rho = 0.47, *p* < 0.01) participants (See Table 4).

### Factor analysis

**Fig. 2:**
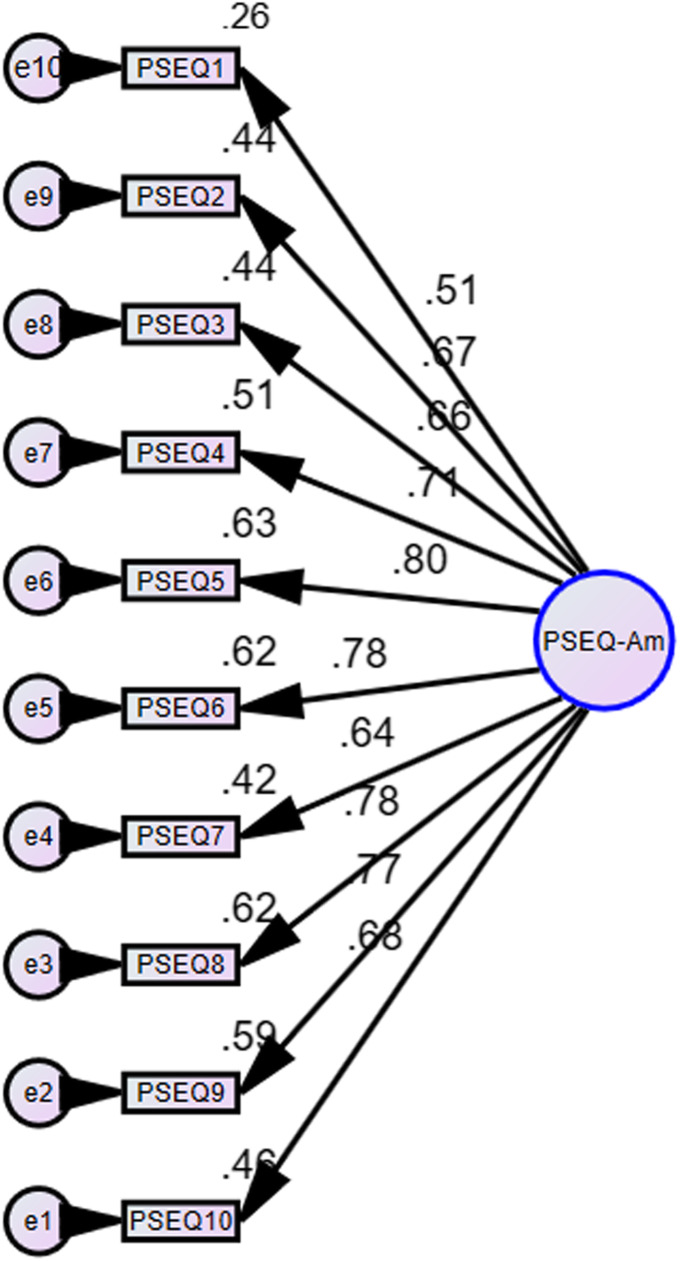
Confirmatory Factor Analysis: Initial Model [(X^2^ (35) =288.45 (*p* < 0.001); X^2^/df = 8.24; CFI = 0.813; PCFI = 0.63; GFI = 0.78; PGFI = 0.5; RMSEA = 0. 17]

The initial CFA and further analysis with modification of indices fail to support a previously hypothesized one-factor model of PSEQ-Am [15, 25] (see Table 6 and Fig. 2). Therefore, Exploratory Factor Analysis (EFA) was performed to determine the dimensionality of PSEQ-Am. The Kaiser Meyer Olkin (KMO) and Bartlett’s criteria with a retention rule of an eigenvalue > 1 were employed to determine the dimensionality of the tool [59]. Besides, a Scree plot was visually inspected. The EFA was conducted using a Maximum Likelihood with an oblique rotation (direct oblimin), and extraction was done using a 0.4-factor loading principle [60, 61].

**Table 6:** A model quality of fitness test for PSEQ-Am using a Confirmatory Factor Analysis

	Chi-Square/ degree of freedom (X^2^/df)	Comparative Fit Index (CFI)	Parsimony Comparative Fit Index (PCFI)	Goodness of Fit Index (GFI)	Parsimony Goodness of Fit Index (PGFI)	Root mean Square Error of Approximation (RMSEA
Initial analysis	288.45/35	0.81	0.63	0.78	0.5	0.17

**Fig. 3:**
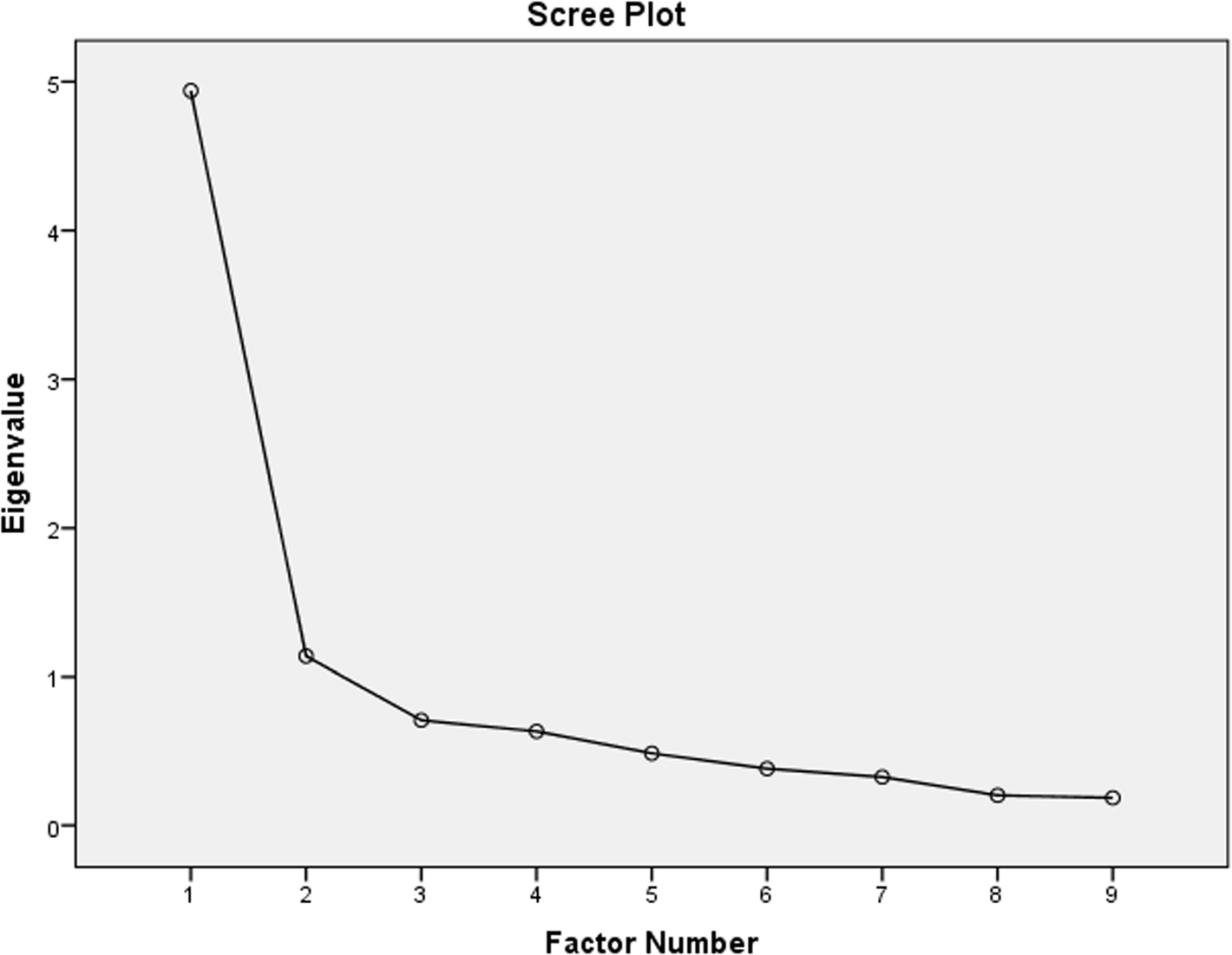
Scree plot indicating factor loading for PSEQ-Am

EFA was conducted as follows. The initial step of EFA with a maximum likelihood using oblique rotation resulted in a two-factor structure (KMO = 0.88 and Chi-square = 1377.02, *p* < 0.001). However, item #4 (“*I can cope with my pain in most situations*”) cross-loaded in both factors (i.e., 0.383 in factor 1 and 0.378 in factor 2). In the next step, we removed item #4 and repeated the analysis using the same procedure, which resulted in KMO = 0.86, Chi-square = 1214.87, degree of freedom = 36, *p* < 0.001. As shown in Table 7 and Fig. 3, PSEQ-Am is predominantly a one-factor and a secondary two-factor structure. The first factor explained the variance of 58.88 with an eigenvalue of 4.939. The two factors explained a total variance of 67.53.

**Table 7:** Factor loading based on Maximum Likelihood with oblimin rotation for 9 items of the PSEQ-Am

Pattern Matrix		
	Factors	
	1	2
PSEQ2	.907	
PSEQ5	.888	
PSEQ3	.654	
PSEQ6	.546	
PSEQ1	.434	
PSEQ9		−.934
PSEQ10		−.797
PSEQ8		−.785
PSEQ7		−.486
The eigenvalue of each factor		
% explained Variance of each factor		

KMO = 0.86

X^2^ = 1214.87***

Only factor loading > 0.3 is indicated, KMO: Kaiser-Meyer-Olkin measure of sampling adequacy; X^2^: Bartlett’s test of sphericity tested with Chi-square; ***p < 0.001; Extraction method: Maximum Likelihood; Rotation Method: Oblimin with Kaiser Normalization

^a^Rotation converged in 8 iterations

## Discussion

This is the first study that cross-culturally translated, adapted, and examined the reliability and validity of the PSEQ in an Ethiopian sample of individuals with LBP.

The results of this study demonstrate that the PSEQ-Am has excellent internal consistency (α = 0.90) and test-retest reliability (ICC = 0.93) (95% Confidence Interval: 0.87–0.95). Our finding is consistent with the results of the original tool for its internal consistency (α = 0.92) [62]. The test-retest reliability is consistent with previous studies of the reliability of the original tool conducted in different settings among people with LBP [63] and people with various pain conditions [8, 15, 24]. However, the finding from the present study is slightly higher than what was reported in the original tool (ICC = 0.79) [62]. The high test-retest reliability among our sample might be related to a possible recall bias as a result of the short time interval between the two tests (3–5 days). In general, the Bland-Altman plot and an excellent ICC value in our study suggest the high test-retest reliability of the Amharic version of PSEQ [21, 23, 64].

The validity of PSEQ-Am was established in two ways. Firstly, we conducted a qualitative cognitive debriefing with a sample of 20 people with LBP to establish the content validity of the tool [65, 66]. Cognitive debriefings were used to assess if the PSEQ-AM captures the important concepts of the construct of interest (pain self-efficacy) and whether the participants understand the concept being measured, the meaning of each item in the tool, and the ease of tool administration [67]. The findings of the cognitive interviews suggest the tool assesses one’s confidence in the ability to perform specific tasks of daily living despite their pain.

Secondly, the construct convergent validity of the tool was assessed by performing a correlation test. As hypothesized, the validity of PSEQ-Am is reflected in its significant moderate correlation (in the expected direction) with the Bodily Pain subscale of the SF-36 tool (Rho = 0.51). Similar to our study, a significant moderate correlation was recorded between PSEQ and Bodily Pain subscale of the SF-36 in previous Japanese study (*r* = 0.45) [6], and Chinese studies (*r* = 0.40 [8] and *r* = 0.56 [22]). The positive correlation between PSEQ-Am and SF-36 Bodily Pain may be explained by a relationship between self-efficacy and other pain-related outcomes such as activity limitations in people with pain conditions [8, 24, 68]. Overall, PSEQ-Am demonstrated an acceptable level of construct validity, as all of our priori hypotheses were met based on criteria suggested in the COSMIN (Consensus-based Standards for the Selection of health status Measurement Instruments) reporting guideline [46, 69].

The PSEQ-Am also showed a significant moderate correlation with the General Health perception of the SF-36. A positive correlation between PSEQ and General Health perception is unsurprising as the total score of pain self-efficacy has positively correlated with higher health-related quality of life in a previous study [25]. For instance, an Iranian study showed the presence of a significant moderate correlation (*r* = 0.52) between General Health and Bodily Pain SF-36 [25].

As can be seen in Table 4, our findings suggested the presence of positive but weak correlations between PSEQ-Am and the Physical Functioning, Role Functioning Physical, Social Functioning, Role Functioning Emotional, Emotional Well-being, and Energy/Vitality subscales of SF-36-Am (Rho< 0.4). The strength of correlation between PSEQ and these subscales varied (weak to moderate) in previous studies among people with different pain conditions [6, 22, 25, 63]. Such variability in the magnitude of correlation may be attributed to the socio-demographic and clinical differences between the study participants. Regardless, as hypothesized, our findings suggest the presence of a significant positive correlation (weak to moderate) between the total score of PSEQ-Am and SF-36-Am subscales.

The presence of moderate positive correlation between PSEQ-Am and the other sub-scales of SF-36: Physical functioning, Bodily Pain, Emotional Wellbeing, and General Health in the disaggregate analysis by the duration of pain provide additional support for the validity of the tool. This finding aligns with previous assumptions that self-efficacy is associated with not only the physical health of an individual living with chronic pain but also their adjustment to the pain [19].

Regardless, the presence of a moderate correlation between PSEQ-Am and SF-36-Am Bodily Pain in both acute (Rho = 0.46) and chronic (Rho = 0.67) samples in our study may indicate that people with higher self-efficacy beliefs can execute activities of daily living despite the pain [8], which confirms our initial hypothesis of the presence of a moderate correlation between PSEQ and Bodily Pain of the SF-36-Am tool. The positive correlation between these two measures may also mean that people with a higher score of bodily pain sub-scale on SF-36-Am have higher pain self-efficacy; as a higher score of Bodily Pain sub-scale on SF-36 indicates lower pain severity and pain interference with activities of daily living. The SF-36 Bodily Pain has two items. The first item assesses pain severity, while the second item assesses the degree to which pain interferes with activities of daily livings [70].

Evidence suggests that there are important clinical and experiential pain differences attributed to sex and gender [71, 72]. While the reliability estimates were high in both men and women, a disaggregated analysis by sex shows that Physical Functioning, Social Functioning, Bodily Pain, Emotional Wellbeing, and General Health Perception of the SF-36 subscales were moderately correlated with PSEQ-Am among men with LBP. However, only Bodily Pain was moderately correlated with PSEQ-Am in women, while the other subscales were only weakly correlated. The mechanism underlying this difference between men and women is unclear. Such a difference in the correlation between PSEQ-Am and SF-36 subscales could be related to sex or gender role differences in pain perception and expression [71, 72]. Future studies involving Rasch analysis to look at differential item functioning (DIF) of the tool based on sex of the participants in the Ethiopian sample of LBP may help to understand these sex or gender differences. Qualitative research may also provide valuable evidence on gender-differences in pain self-efficacy in Ethiopia.

The present study did not support a previously suggested unidimensionality of the PSEQ [15, 19, 23, 25]. As shown in Table 6, the explained variance of the first structure is four times that of the second factor, although both factors demonstrated eigenvalues > 1. Furthermore, a visual inspection of the scree plot suggests of a dominant one-factor structure of the tool with the addition of a second factor. The reason why PSEQ-Am failed to conform to a previously hypothesized one-factor structure is unclear. However, PSEQ-Am may measure pain self-efficacy with other underlying constructs among people with LBP in Ethiopia which require further investigation. As discussed in previous literature, several psychosocial and cultural factors may affect one’s perception of abstract concepts such as pain [73] and self-efficacy [71]. Future studies are needed to evaluate psycho-social and cultural factors which may influence pain self-efficacy constructs among people with LBP in Ethiopia.

Floor and ceiling effect occurs when more than 15% of participants reported the highest or lowest possible score on a survey tool [45]. Our finding suggests that PSEQ-Am does not have either floor or ceiling effect, which is in line with a previous study [21]. The lack of floor and ceiling effect in PSEQ-Am shows that the tool may be able to measure the entire spectrum of self-efficacy among Amharic speaking people with LBP in in Ethiopia.

In general, the findings from our study suggest that the Amharic version of the Pain Self-Efficacy Questionnaire has adequate validity and reliability and warrants its use in clinical settings to assess the level of pain self-efficacy among people with LBP and the outcome of pain management among Ethiopian people with LBP. In addition, the availability of a validated PSEQ-Am is useful for research evaluating pain self-efficacy studies among people with LBP in Ethiopia. Furthermore, the tool can also be beneficial in the understanding of cross-cultural similarities and differences in the role of pain self-efficacy in the rehabilitation of Ethiopian low back pain patients and similar patient population across other settings.

This study has a few limitations. Due to the absence of other validated pain measures, such as the Brief Pain Inventory pain interference scale [19, 74] in Ethiopian settings, we used the SF-36-Am tool to validate PSEQ-Am. Hence, we were not able to do every possible type of validity (e.g., discriminant validity), suggesting a need for further psychometric tests of this tool in future studies by comparing it with self-efficacy scales. Furthermore, due to the absence of other validated measures such as Global Rating of Change Scales (GRCS) which serves as anchor [75, 76] in Ethiopian context, we were not able to differentiate improved and stable group of people with LBP as a result of the intervention they were receiving or clinical course of the condition during data collection. Additionally, this study did not assess the degree to which PSEQ-Am is sensitive to pain management changes in our sample population. Future research is needed to assess whether the measure of pain self-efficacy changes in response to LBP treatment in Ethiopia.

## Conclusion

The Amharic version Pain Self-efficacy Questionnaire has strong psychometric properties among a sample of people with LBP in Ethiopia. PSEQ-Am demonstrated an excellent test-retest reliability and internal consistency, and convergent construct validity with Bodily Pain of SF-36-Am. The translation, adaptation, and validation of this tool in Amharic provides a tool for assessing self-efficacy in clinical practice or research with people with LBP in Ethiopia.

## Supplementary Information

**Additional file 1: Appendix 1.** The final version of the PSEQ-Am.

## Data Availability

Data for this study is available and can be provided by the corresponding author upon request.

## Abbreviations

Amos: Analysis of Moment Structures
CFA: Confirmatory Factor Analysis
ICC: Intraclass correlation coefficient
LBP: Low back pain
PSEQ: Pain Self-Efficacy Questionnaire
PSEQ-Am: Pain Self-Efficacy Questionnaire Amharic version
Rho: Spearman’s Correlation coefficients
SF-36: Short Form Health Survey
SF-36-Am: Short Form Health Survey Amharic version
SPSS: Statistical Package for the Social Sciences
