# Supplementary material for: Cross-cultural translation, adaptation, and validation of the Amharic version pain self-efficacy questionnaire in people with low back pain in Ethiopia
Source: BMC Musculoskelet Disord. 2021 Jan 25;22:111. doi: 10.1186/s12891-021-03985-4 (PMC7836442; doi:10.1186/s12891-021-03985-4)
Supplement: Supplementary file 1 — Additional file 1: Appendix 1. The final version of the PSEQ-Am. [file 12891_2021_3985_MOESM1_ESM.pdf]

<u>ፔይን ሰልፍ ኤፍከሲ መጠይቅ (Pain Self-efficacy questionnaire)</u>

እባክዎ ከዚህ በታች የተዘረዘሩትን ተግባራት ህመም ቢኖርም በምን ያህል በራስ መተማመን ማድረግ እንደሚችሉ ይግለጹ።መልስዎን በእያንዳንዱ ተግባራት ትይዩ በተቀመጡት መመዘኛዎች ላይ ያክቡ። መልስዎን ለመስጠት በቁጥር ከ ዜሮ እስከ ስድስት ካሉት ቁጥሮች ውስጥ ኣንዱን ብቻ በማክበብ ይግለጹ፡ ይሀዉም ዜሮ ማለት በፍጹም በራስ መተማመን የለኝም ሲሆን ስድስት ማለት ደግሞ ሙሉ በሙሉ በራስ መተማመን ኣለኝ ማለት ነው።

ያስታውሱ፤ ከዚህ በታች የተዘረዘሩትን ነገሮች ከዚህ በፊት ማከናወን እንደሚችሉ እና እንደማይችሉ የሚጠይቅ ሳይሆን በአሁኑ ወቅት ምንም እንኳን ህመም ቢኖርም በምን ያህል የራስ መተማመን ማከናወን እንደሚችሉ ብቻ ነው።

	በጭራሽ አልተማመንም						በጣም እተማመናለሁ
1. ህመም ቢኖርም እንኳን በነገሮች እደሰታለሁ	0	1	2	3	4	5	6
2. ሕመም ቢኖርም እንኳን አብዛኛውን የቤት ውስጥ ስራዎችን መስራት እችላለሁ (ለምሳሌ ቤት ማፅዳት፣ ዕቃ ማጠብ ፣ ኣልጋ ማንጠፍ ፣ እቃ ማንሳት፣ ምግብ ማብሰል፣ እንጨት መፍለጥ፤ ወዘተ)::	0	1	2	3	4	5	6
3. ሕመም ቢኖርብኝም እንኳን ሁልጊዜ እንደማደርገው ከጓደኞቼ ወይም ከቤተሰቤ ጋር ማህበራዊ ግኑኝነት ማድረግ እችላለሁ::	0	1	2	3	4	5	6
4. በ ብዙ ሁኔታዎች ህመሜን መቋቋም እችላለሁ	0	1	2	3	4	5	6
5. ህመም ቢኖርም አንዳንድ ስራዎችን መስራት እችላለሁ ("ስራዎቹ" የቤት ውስጥ ፣ ክፍያ ያለው እና የሌለውም ሊሆን ይችላል)::	0	1	2	3	4	5	6
6. ህመም ቢኖርብኝም እንኳን ለመዝናናት ወይም በትርፍ ጊዜየ የማከናውናቸው በርካታ ተግባራት አሁንም ኣደርጋቸዋለሁ፡፡	0	1	2	3	4	5	6
7. መድሃኒት ሣልወስድ ህመሜን መቋቋም እችላለሁ።	0	1	2	3	4	5	6

8. ህመም ቢኖርም እንኳን አብዛኛዎቹን የህይወት አላማዬን ማሳካት እችላለሁ፡፡	0	1	2	3	4	5	6
9. ህም ቢኖርም እንኳን መደበኛ ህይወቴን መምራት እችላለሁ፡፡	0	1	2	3	4	5	6
10. ህመም ቢኖርም እንኳን ቀስ በቀስ የበለጠ በአካል ንቁ መሆን እችላለሁ፡፡	0	1	2	3	4	5	6
